# Efficacy of topical Ivermectin in controlling human *Demodex* infestation: Evidence from systematic review and meta-analysis

**DOI:** 10.1016/j.parepi.2025.e00461

**Published:** 2025-09-23

**Authors:** Anon Paichitrojjana, Kitsarawut Khuancharee, Anand Paichitrojjana

**Affiliations:** a School of Anti-Aging and Regenerative Medicine, Mae Fah Luang University, Bangkok, Thailand; b Department of Preventive and Social Medicine, Faculty of Medicine, Srinakharinwirot University, Nakhon Nayok, Thailand; c Faculty of Medicine, Ramathibodi Hospital, Mahidol University, Bangkok, Thailand

**Keywords:** Topical ivermectin, *Demodex* mite, Rosacea treatment, Meta-analysis, Skin health, Evidence-based dermatology

## Abstract

**Background:**

*Demodex* mites are usually harmless but can contribute to inflammatory skin conditions like rosacea, blepharitis, and demodicosis. While new therapies like lotilaner, niclosamide, and berberine show promise against *D. folliculorum*, ivermectin remains widely used for its strong antiparasitic and anti-inflammatory effects. However, the direct effectiveness of ivermectin in reducing *Demodex* mite density in associated skin diseases is not fully quantified.

**Materials and methods:**

A systematic review and meta-analysis were conducted following PRISMA guidelines. PubMed, Scopus, the Cochrane Library, and Google Scholar were searched for studies published between December 2014 and December 2024. Eligible studies have assessed the effect of topical ivermectin on *Demodex* mite number or density using standardized diagnostic methods. Risk of bias was evaluated using the Risk of Bias Assessment Tool for Non-randomized Studies (RoBANS) and the Cochrane Risk of Bias Tool. Data were pooled using a random-effects model where appropriate.

**Results:**

A total of 2344 studies were identified, with five studies (*n* = 180 participants) meeting the inclusion criteria. All studies reported significant reductions in *Demode*x mite count or density after daily application of topical ivermectin 1 %. Meta-analyses demonstrated a mean reduction of 70.01 mites/cm^2^ and an 80 % decrease in *Demodex*-positive (≥5 D/cm^2^) rates. A 16-week treatment duration was associated with a notable reduction, with effects sustained for up to 12 weeks post-treatment. Only mild, localized adverse events were reported, with no systemic side effects observed.

**Conclusion:**

Topical ivermectin is effective and well-tolerated for reducing the number and density of *Demodex* mites. A 16-week treatment course significantly decreases mite burden and improves clinical outcomes with minimal adverse events. However, the potential for mite repopulation after treatment underscores the importance of ongoing monitoring. Study heterogeneity and the limited number of included trials warrant cautious interpretation of the findings.

## 1 Introduction

*Demodex* mites are microscopic ectoparasites residing on the skin of many mammals, including humans. Two species, *Demodex folliculorum* and *Demodex brevis*, commonly inhabit hair follicles and sebaceous glands, respectively (Paichitrojjana, 2022). While typically harmless in small numbers, an overgrowth of *Demodex* mites has been linked to several inflammatory skin conditions (Paichitrojjana and Paichitrojjana, 2025). The precise role of *Demodex* in these diseases remains under investigation, but growing evidence suggests that they contribute to skin inflammation (Badescu et al., 2013; Wei et al., 2024). This emphasizes the need for effective treatments to reduce mite populations and mitigate associated symptoms (Forton, 2020).

Rosacea is among the most extensively studied conditions related to *Demodex* overgrowth. Characterized by facial erythema, papules, pustules, and telangiectasia, rosacea affects approximately 5.5 % of the global population, with higher prevalence among individuals of European descent (Gallo et al., 2018). Studies have demonstrated a correlation between increased mite density and rosacea severity, likely mediated by host immune dysregulation (Forton, 2020). Similarly, *Demodex* infestation is implicated in the pathogenesis of blepharitis, contributing to eyelid inflammation and meibomian gland dysfunction (Rhee et al., 2023). In some cases, uncontrolled mite proliferation can lead to overt demodicosis (Paichitrojjana, 2022).

Recent years have introduced new treatments for *Demodex* blepharitis. Lotilaner, a selective GABA chloride channel inhibitor, shows high mite eradication rates (Gaddie et al., 2023; Muhammad Muneeb Akhtar et al., 2024). Niclosamide and berberine also demonstrate potent in vitro activity against *D. folliculorum*, sometimes outperforming tea tree oil, with good safety profiles (Guo et al., 2025; Li et al., 2025). However, ivermectin remains commonly used in clinical practice.

Topical ivermectin has emerged as a leading treatment due to its dual antiparasitic and anti-inflammatory effects. It paralyzes and kills mites by binding glutamate-gated chloride channels and attenuates inflammation by suppressing pro-inflammatory cytokine production. In 2014, the U.S. Food and Drug Administration (FDA) approved 1 % ivermectin cream for the treatment of papulopustular rosacea following clinical trials that demonstrated its efficacy over placebo. (Raedler, 2015) However, while symptom improvement is well established, the direct impact of ivermectin on *Demodex* number and density remains less clear.

Objective methods such as standardized skin surface biopsy (SSSB) and reflectance confocal microscopy (RCM) enable precise quantification of mite populations, providing an opportunity to objectively assess treatment outcomes. (Forton and Seys, 1993; Sattler et al., 2015) Research assessing the impact of ivermectin on *Demodex* levels is limited and methodologically heterogeneous. While previous systematic reviews have examined a range of anti-*Demodex* therapies, including ivermectin, tea tree oil, permethrin, metronidazole, and systemic agents, there is still no focused synthesis that offers high certainty regarding the efficacy of topical ivermectin (Li et al., 2023).

This systematic review and meta-analysis aimed to assess the effectiveness and safety of topical ivermectin in reducing both the number and density of *Demodex* mites. By concentrating on the objective measurement of mite burden, this study seeks to offer clear, evidence-based guidance for managing skin diseases associated with *Demodex*.

## 2 Materials and methods

### 2.1 Searching strategy

A comprehensive literature search was conducted across multiple electronic databases, including PubMed, Scopus, the Cochrane Library, and Google Scholar, to identify relevant studies examining the efficacy and safety of topical ivermectin in reducing *Demodex* mite infestations. The search period spanned from December 26, 2014, to December 26, 2024. We employed the following search formula: ([*Demodex*] OR [demodicosis]) AND (ivermectin) AND (topical). Our focus was on articles that explore the relationship between *Demodex* mites and the use of topical ivermectin. This targeted approach aimed to capture various studies to support our research objectives.

### 2.2 Study selection and screening process

Following database searches, all retrieved citations were imported into EndNote for reference management, and duplicate records were systematically removed. Two independent reviewers, blinded to each other's decisions, screened the titles and abstracts to assess the relevance of the studies based on predefined eligibility criteria. Studies considered potentially relevant underwent a full-text review for final inclusion. Any disagreements regarding study selection were resolved through consensus or consulting a third independent reviewer. A PRISMA (Preferred Reporting Items for Systematic Reviews and Meta-Analyses) flow diagram documented the screening process, including the reasons for any study exclusions. The screening was carried out using a standardized eligibility checklist developed in advance.

### 2.3 Eligibility criteria

#### 2.3.1 Inclusion criteria

The inclusion criteria focus on clinical research that evaluates the efficacy and safety of topical ivermectin for treating demodicosis and other conditions associated with *Demodex*. To be included, studies must report measurable outcomes related to the reduction of *Demodex* number or density, utilizing standardized diagnostic tools such as the SSSB, microscopy, and RCM.

#### 2.3.2 Exclusion criteria

Exclusion criteria include case reports, editorials, research letters, letters to the editor, reviews, animal studies, basic biomedical research, non-human medicine studies, and inaccessible full texts.

### 2.4 Quality assessment

Two reviewers independently verified the data for accuracy. Any discrepancies in quality assessment were resolved through discussions with a third reviewer. The Risk of Bias Assessment Tool for Non-Randomized Studies (RoBANS) was used to evaluate the methodological quality of the studies, which included a six-item assessment for each case series (Kim et al., 2013). If randomized controlled trials were included, the Cochrane Risk of Bias Tool was applied. Sensitivity analyses were performed to assess the impact of study quality on the pooled results.

### 2.5 Data extraction

Two independent reviewers performed data extraction using a standardized form. The following information was collected from eligible studies: first author, publication year, country, study design, sample size, population characteristics (e.g., age, gender, and baseline clinical severity), intervention details, adverse events, and reported outcomes, and statistical analysis (key findings, confidence intervals, and *p*-values). Discrepancies in data extraction were resolved through consultation with the third reviewer. One reviewer entered all extracted data into Review Manager 5. Two reviewers independently verified the accuracy of the data entry. In cases where data were incomplete, attempts were made to contact the corresponding authors of the original studies for clarification.

### 2.6 Data analysis

Meta-synthesis was conducted using the Review Manager web tool. For data presented as medians and interquartile ranges (IQR), means and standard deviations (SD) were estimated using methods developed by Wan et al. (2014); Luo et al. (2018). The mean difference was used for continuous outcomes, while *Demodex* reduction was applied for categorical outcomes using the inverse-variance method. Heterogeneity among studies was assessed using Cochran's Q test (χ^2^), with statistical significance set at *p* < 0.10. The I^2^ statistic was used to quantify heterogeneity, categorized as 0 % (no heterogeneity), 25 % (low), 50 % (moderate), and 75 % (high) (Moher et al., 2009). A fixed-effect model was applied when no significant heterogeneity was detected (*p* > 0.10). In cases where heterogeneity was present (*p* < 0.10) with moderate or higher I^2^ values (≥50 %), a random-effects model was used (Higgins et al., 2003).

This review was registered in the PROSPERO database (Registration ID: CRD420251017433) and conducted in accordance with the PRISMA 2020 guideline.

## 3 Results

### 3.1 Search strategy

**Fig. 1:**
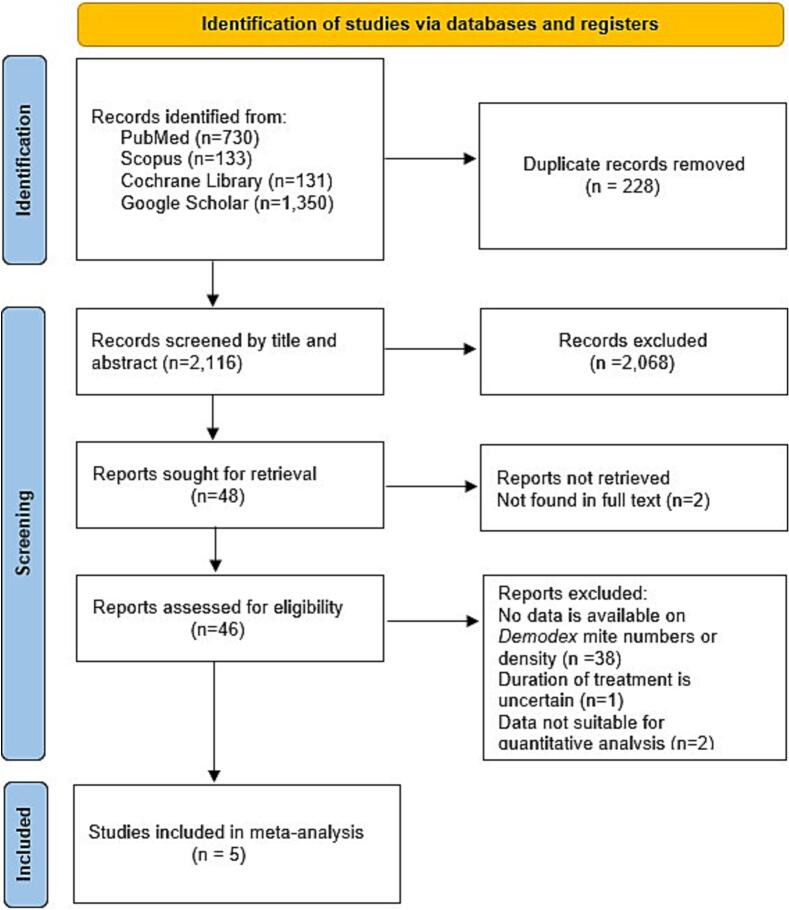
PRISMA flow diagram illustrating the study selection process.

The systematic database search results are shown in Fig. 1. A total of 2344 records were initially retrieved from PubMed, Scopus, Cochrane Library, and Google Scholar. After removing 228 duplicates, 2116 unique records remained for screening. Of these, 2068 studies were excluded based on titles and abstracts for not meeting the inclusion criteria, leaving 48 full-text articles for eligibility assessment. Two reports could not be retrieved. Following full-text review, 38 articles were excluded due to the absence of data on *Demodex* mite numbers or density, one study was excluded for uncertain treatment duration, and two studies were excluded due to unsuitable data for quantitative analysis. Ultimately, five studies met all inclusion criteria and were included in the meta-analysis (Trave et al., 2022; Katsitadze et al., 2024; Logger et al., 2022; Schaller et al., 2017; Trave et al., 2019).

### 3.2 Study characteristics

The five included studies are summarized in Table 1. Published between 2017 and 2024, these studies evaluated the efficacy of 1 % topical ivermectin in treating *Demodex*-associated rosacea, with a focus on papulopustular rosacea (PPR). They involved both retrospective and prospective designs, with sample sizes ranging from 20 to 60 participants, and were conducted across Europe and Western Asia. All studies used a once-daily application regimen for 12 to 16 weeks, with one study including a 32-week post-treatment follow-up to assess relapse rates. *Demodex* mite numbers and densities were assessed using various methodologies, including light microscopy of skin scrapings, SSSB, and RCM. All studies reported a reduction in *Demodex* mite counts or densities, with significant decreases consistently observed following the daily topical ivermectin application.

**Table 1:** Information on the included studies regarding the efficacy and safety of topical ivermectin.

Study	Study design	Diagnosis	Sample size	Age (Mean ± SD)	% Male	Intervention	Follow-up duration	Methods to detect *Demodex* mites	Efficacy	Safety	Risk of Bias
Trave et al., 2019	Retrospective Study	Rosacea	50 (16 positive for mites)	56 ± 14.7	25	Topical ivermectin 1% once daily for 16 weeks	16 weeks	SSSB; Every sample with ≥5 D/cm^2^ was considered positive	The outcomes were excellent in 54 %, good 36 %, and moderate 6 % -*Demodex* positivity reduced from 16 to 0	0 % had none-to-mild adverse events, 6 % had severe reactions	High
Trave et al., 2022	Prospective Study	Rosacea	60 (20 positive for mites)	57 ± 11.2	25	Topical ivermectin 1% once daily for 16 weeks	32 weeks	SSSB; Every sample with ≥5 D/cm^2^ was considered positive	-Success rate for *Demodex* infestation was 87.5%, with a 12.5 % relapse-*Demodex* positivity reduced from 20 to 4	None reported	Low
Schaller et al., 2017	Prospective Study	Rosacea	20	52.3 ± 13.1	40	Topical ivermectin 1% once daily for at least 12 weeks	≥12 weeks	Two consecutive SSSB; Demodex mite density (D/cm^2^) (mean ± SD)	-The mean IGA score had significantly decreased (P < 0.001) -*Demodex* density at baseline was 99.9 ± 16.75 fell to 3.8 ± 0.96 at ≥6 weeks, and 0.8 ± 0.31 at ≥12 weeks	Patients reported no adverse events	Moderate

Study	Study design	Diagnosis	Sample size	Age (Mean + SD)	% Male	Intervention	Follow-up duration	Methods to detect Demodex mites	Efficacy	Safety	Risk of Bias
Logger et al., 2022	Prospective Study	Rosacea	20 (16 positive for mites)	52.8 ± 14.5	45	Topical ivermectin 1% once daily for 16 weeks	28 weeks	Reflectance Confocal Microscopy examinations for *Demodex* mites	Inflammatory lesions significantly decreased from baseline by week 12 and continued to decline by week 16-Mites detected baseline = 13 (0.0–203), week 12 = 0 (0.0–10), week 16 = 0 (0.0–14) and week 28 = 1(0.0–21)	Eleven patients reported mild skin adverse events: stinging/burning, facial erythema, itching, and dryness. All were self-limiting	Moderate
Katsitadze et al., 2024	Prospective Study	Rosacea	30	40 (Range: 30–50)	33.3	Group I: Topical ivermectin 1% Group II: Topical emtronidazole 1% for 12 weeks	12 weeks	Microscopic examination of skin scrapings	Ivermectin reduced *Demodex* density and erythema severity more effectively than metronidazole-The presence of mites in the smear has diminished and, in some cases, can no longer be detected	No severe adverse effects were reported.-Minimal skin irritation in some cases	Moderate

SSSB, Standardized Skin Surface Biopsy; IGA, Investigator Global Assessment; SSSB, Standardized Skin Surface Biopsy.

### 3.3 Risk of bias assessment

**Fig. 2:**
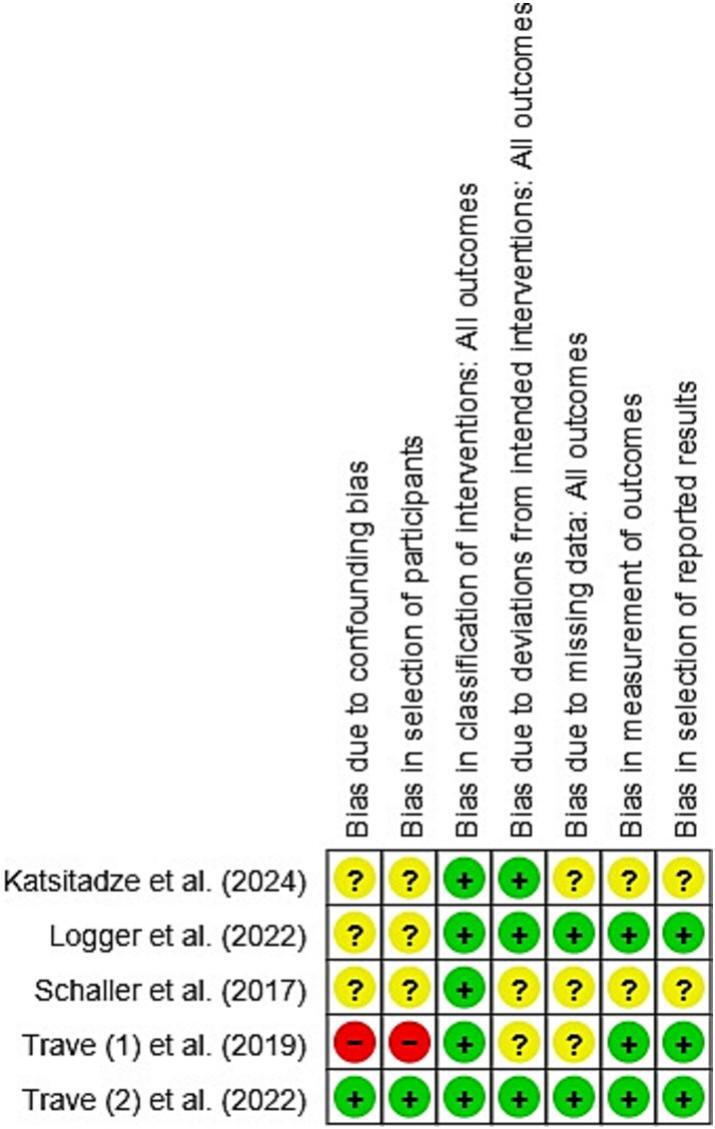
Methodological quality of included cohort articles.

**Fig. 3:**
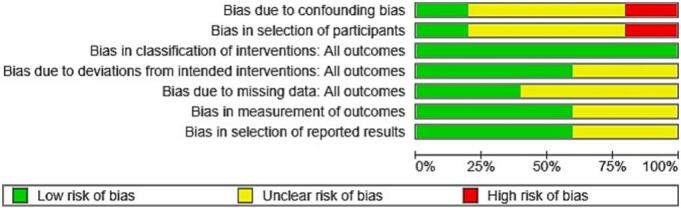
Summary bias risk of included cohort studies articles.

The risks of bias assessments for the studies included are shown in Fig. 2, Fig. 3. Most studies demonstrated a low to moderate risk of bias in domains such as reported results selection and outcome measurement. However, some studies lacked clarity regarding participant selection and confounding control, which may have influenced internal validity.

### 3.4 Quantitative synthesis: Meta-analyses

#### 3.4.1 Reduction in *Demodex* mite number

**Fig. 4:**
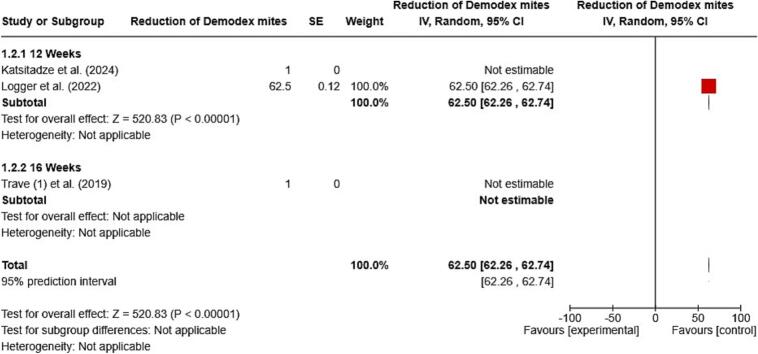
Meta-analysis on the effectiveness of topical ivermectin in reducing *Demodex* mite populations (95 % CI: 95 % confidence intervals).

The meta-analysis was conducted to assess the effectiveness of topical ivermectin treatment in reducing *Demodex* mites in patients. The pooled proportion analysis indicated that a significant percentage of patients experienced a reduction in *Demodex* mites, with a rate of 62.50 % (95 % Confidence Intervals (CI): 62.26 to 62.74) after 12 weeks of treatment. The overall treatment effect was highly significant, with a *Z*-score of 520.83 and a *p*-value less than 0.00001 (see Fig. 4).

#### 3.4.2 Reduction in *Demodex* mite density

**Fig. 5:**
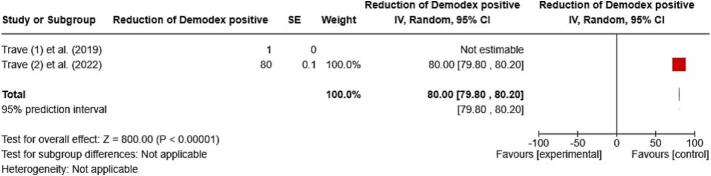
Meta-analysis on reduction of *Demodex* mite positive (≥5 mites/cm^2^) rate in topical ivermectin (95 % CI: 95 % confidence intervals).

Meta-analyses provided data that allowed for an analysis of the post-treatment rate of *Demodex* positivity (≥5 D/cm^2^). The pooled proportion analysis indicated a significant decrease, with a post-treatment positive rate of 80.00 % (95 % CI: 79.80–80.20) after 16 weeks of treatment. The treatment effect was highly significant (Z = 800.00, *P* < 0.00001) (see Fig. 5).

#### 3.4.3 Time-dependent analysis

**Fig. 6:**
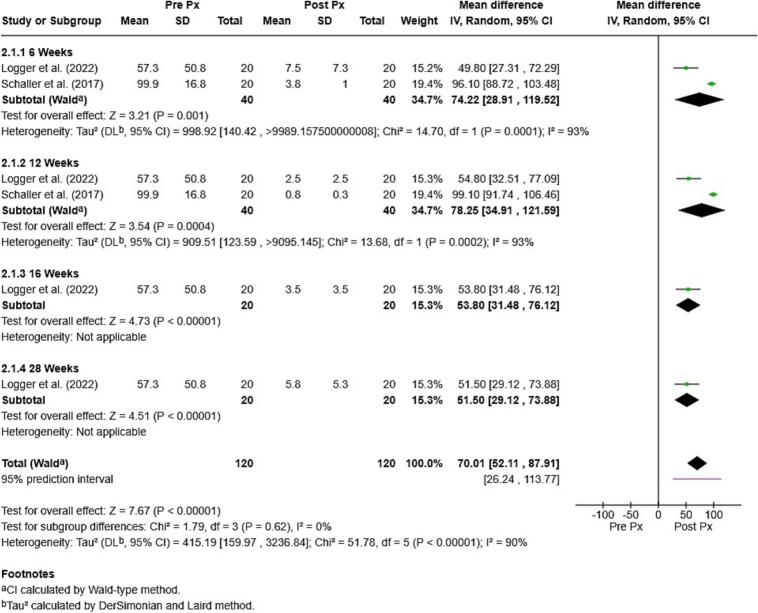
Meta-analysis on mean difference of *Demodex* mites before and after treatment in topical ivermectin (95 % CI: 95 % confidence intervals).

A meta-analysis examining the mean difference in *Demodex* mite counts before and after topical ivermectin treatment demonstrated a substantial reduction. The pooled mean difference was 70.01 (95 % CI: 52.11–87.91), indicating a significant treatment effect. Subgroup analysis based on treatment duration showed that mite reduction began as early as week 6 and was sustained through week 16. A decrease in mite counts remained evident during the 28-week follow-up (12 weeks post-treatment), although the reduction was slightly attenuated. However, considerable heterogeneity was observed across the included studies (I^2^ = 90 %) (see Fig. 6).

#### 3.4.4 Adverse events

All included studies reported low rates of adverse effects, primarily consisting of mild and localized skin reactions. No systemic adverse events or treatment discontinuations were observed. A summary of the adverse events is presented in Table 1.

## 4 Discussion

Ivermectin, a broad-spectrum antiparasitic derived from *Streptomyces avermitilis*, acts on glutamate-gated chloride channels in invertebrates, causing paralysis and death. Initially used in veterinary medicine, it is now widely employed to treat human parasitic diseases (Burg et al., 1979; Campbell and Benz, 1984; Crump and Ōmura, 2011; Kaur et al., 2024). Oral ivermectin effectively targets *Demodex* mites, reducing both mite density and clinical symptoms. However, its short half-life and lack of ovicidal activity require extended treatment durations (Li et al., 2023; Truchuelo-Díez et al., 2011; Holzchuh et al., 2011; Paichitrojjana and Chalermchai, 2024). Topical 1 % ivermectin cream has shown efficacy in papulopustular rosacea and *Demodex* blepharitis, with minimal adverse effects (Gupta et al., 2015; Gazewood and Johnson, 2016; Choi et al., 2022). However, most studies emphasize symptom resolution over mite count reduction, underscoring the need for a comprehensive approach that considers both clinical and parasitological outcomes.

### 4.1 Efficacy of 1 % topical ivermectin in *demodex*-associated dermatoses

This systematic review and meta-analysis support the effectiveness of 1 % topical ivermectin in reducing *Demodex* mite, inflammation, and improving clinical outcomes, particularly in papulopustular rosacea.

All five included studies reported significant symptom improvement after 12–16 weeks of once-daily application, with minimal adverse effects. Trave et al. (2022) reported a complete response rate of 70 % with an Investigator Global Assessment (IGA) score of 0 after 16 weeks (Trave et al., 2022). In a comparative trial, ivermectin outperformed metronidazole, with 80 % of patients showing significant improvement versus 33.3 % in the metronidazole group (Katsitadze et al., 2024). Inflammatory lesions in papulopustular rosacea were reduced by 76 % to 97 %, with statistically significant improvements (*p* < 0.001) across all severity levels (Trave et al., 2019).

Relapses occurred in 45.2 % of patients after a median of 140 days following 16 weeks of treatment, indicating the possible benefits of maintenance therapy (Trave et al., 2022). Another study observed sustained clinical improvement 12 weeks after completing a 16-week course, despite partial *Demodex* recolonization (Logger et al., 2022).

Two studies examined the molecular effects of topical ivermectin. Schaller et al. reported significant downregulation of inflammatory markers (LL-37, HBD3, TLR4, TNF-α) at both mRNA and protein levels (*p* < 0.05) (Schaller et al., 2017). In contrast, Logger et al. observed clinical improvement without detectable histological and inflammatory cell population changes on RCM, likely due to imaging limitations (Logger et al., 2022).

Parasitological data showed marked reductions in mite number and density with once-daily 1 % topical ivermectin over 12–16 weeks. Schaller et al. reported a decrease from 99.9 to 3.8 D/cm^2^ by week 6 and further to 0.8 D/cm^2^ by week 12 (*P* < 0.001), indicating rapid and sustained antiparasitic activity (Schaller et al., 2017). Logger et al. found a reduction in mite detection from 80 % to 30 % by week 16, assessed by RCM; however, *Demodex* reemergence was noted in 63 % of patients by week 28 (Logger et al., 2022). Katsitadze et al. showed greater mite reduction with ivermectin compared to metronidazole (Katsitadze et al., 2024). A retrospective analysis by Trave et al. (2019) reported outcomes, with all *Demodex*-positive (≥5 D/cm^2^) patients achieving clearance after 16 weeks (Trave et al., 2019). Trave et al. (2022) confirmed similar outcomes, with 80 % of *Demodex*-positive patients becoming negative by week 16, with only 12.5 % relapsing over 32 weeks (Trave et al., 2022).

### 4.2 Meta-analysis of the efficacy of topical ivermectin in reducing the number and density of *Demodex mites*

A meta-analysis included three studies: Katsitadze et al. (2024), Logger et al. (2022), and Trave et al. (2019). However, only Logger et al. provided analyzable data, which indicated that 62.5 % of patients experienced a reduction in mites after 12 weeks. In contrast, the other two studies reported a 100 % reduction in mites after 12 and 16 weeks, respectively, but their data could not be included in the pooled estimate due to the absence of variance measures.

Another meta-analysis confirmed a significant decrease in *Demodex* density. Trave et al. (2022) reported an 80 % reduction in *Demode*x-positive patients after 16 weeks, which contributed fully to the pooled estimate. In a previous study, Trave et al. (2019) observed a complete 100 % reduction in *Demodex*-positive patients; however, this study was excluded from the meta-analysis because it lacked standard error values.

Additionally, a separate meta-analysis evaluated mite reduction over multiple time points during and after a 16-week treatment course, with evaluations at weeks 6, 12, 16, and 28. The pooled analysis showed a mean reduction of 70.01, demonstrating strong and sustained efficacy with no significant subgroup differences despite substantial heterogeneity (I^2^ = 90 %). During treatment, mite counts declined significantly by week 6 (mean reduction: 74.22), peaked at week 12 (78.25), and remained substantially reduced at weeks 16 (53.80) and 28 (12 weeks post-treatment) (51.50). These results indicate rapid and sustained effectiveness, with partial mite repopulation after treatment likely due to ivermectin's lack of ability to kill eggs. Factors related to the host may also play a role in the risk of relapse.

According to these trends, a 16-week treatment course is practical for achieving a significant reduction in mites and ensuring prolonged remission. This regimen covers multiple life cycles of *Demodex,* which may help eradicate leftover mites and eggs, decrease the chances of early reinfestation, and extend the period of remission. While some repopulation was observed during follow-up, recurrence was notably slower after the 16-week treatment compared to shorter courses. This treatment duration aligns with clinical response timelines from previous studies. A 12-week course has been shown to improve moderate-to-severe papulopustular rosacea, achieving clear or nearly clear skin in 38–40 % of patients, with improvements evident as early as week 4 (Ebbelaar et al., 2018). Extending therapy to 16 weeks increases response rates to 70 % and reduce relapse risk, supporting its use in routine management (Trave et al., 2022).

Topical ivermectin 1 % cream was generally well tolerated across all included studies, with adverse events limited to mild, transient symptoms such as stinging, burning, and itching, predominantly occurring during the initial weeks of treatment. These symptoms are typically resolved without necessitating treatment discontinuation, and no systemic adverse effects were reported. (Logger et al., 2022; Trave et al., 2019) Katsitadze et al. found no significant difference in adverse event rates between ivermectin and metronidazole (Katsitadze et al., 2024). Furthermore, Trave et al. (2022) highlighted the appropriateness of ivermectin for long-term use and maintenance therapy (Trave et al., 2022).

The high heterogeneity observed in the pooled mean difference analysis of this study is likely due to variations in diagnostic methods, baseline mite densities, treatment durations, and follow-up periods, as well as incomplete reporting in some studies. This variability requires cautious interpretation of the findings. However, the consistent trend across all studies, showing significant reductions in *Demodex* burden and clinical symptoms, supports the effectiveness of 1 % topical ivermectin as a treatment option. However, focusing solely on symptom improvement may overlook ongoing or recurrent infestations. Implementing standardized mite monitoring during and after treatment could aid in early relapse detection and guide personalized long-term management.

### 4.3 Limitations of the study

This meta-analysis included only five studies with small sample sizes, limiting the generalizability of the findings. Most used before-and-after designs without control groups, increasing the risk of bias. High heterogeneity was observed, likely due to variations in methodologies. Incomplete data hampered analyses, with effect sizes mainly derived from one study, reducing robustness. Short follow-up periods made it difficult to assess long-term outcomes, and inconsistent reporting of adverse events complicated comparisons. The limited number of studies prevented a formal assessment of publication bias, as guidelines suggest this requires at least ten studies.

## 5 Conclusion

Although substantial heterogeneity, the small sample size, and incomplete data limit quantitative precision, the consistent trend toward efficacy across diverse settings supports the clinical utility of 1 % topical ivermectin for managing *Demodex-*associated dermatoses. While treatment results are generally positive, it is essential to monitor potential mite repopulation. Future large-scale, well-designed trials are needed to validate these results, establish optimal treatment duration, and assess long-term effectiveness and safety.

## Funding

This article has no funding source.

## Ethical considerations

This study adhered to the guidelines outlined in the PRISMA (Preferred Reporting Items for Systematic Reviews and Meta-Analyses) statement. As a secondary analysis of previously published studies, no institutional review board (IRB) approval or patient consent was required.

## CRediT authorship contribution statement

**Anon Paichitrojjana:** Writing – review & editing, Writing – original draft, Visualization, Validation, Supervision, Software, Resources, Project administration, Methodology, Investigation, Funding acquisition, Formal analysis, Data curation, Conceptualization. **Kitsarawut Khuancharee:** Writing – review & editing, Writing – original draft, Visualization, Validation, Supervision, Software, Formal analysis. **Anand Paichitrojjana:** Writing – review & editing, Writing – original draft, Validation, Methodology, Investigation, Data curation.

## Declaration of competing interest

The authors declare that they have no known competing financial interests or personal relationships that could have appeared to influence the work reported in this paper.
